# Ileocecal Valve Metastasis Inducing Cecal Volvulus in a Patient With Lobular Breast Cancer: A Rare Cause of Bowel Obstruction

**DOI:** 10.7759/cureus.73345

**Published:** 2024-11-09

**Authors:** Leonor Ávila, Tiago Branco, Helena Leandro, Rita Silva, Rui Mendes, Fátima Coelho

**Affiliations:** 1 General Surgery, Unidade Local de Saúde de Lisboa Ocidental, Lisbon, PRT; 2 General Surgery, Hospital de Caldas da Rainha, Caldas da Rainha, PRT

**Keywords:** bowel obstruction, cecal volvulus, emergency abdominal surgery, general surgery and breast cancer, hormonal therapy, invasive lobular breast carcinoma, metastatic breast cancer, right-sided hemicolectomy

## Abstract

Invasive lobular carcinoma (ILC) is the second most prevalent form of invasive breast cancer. Characterized by its insidious growth and distinctive histopathological features, ILC often presents with a less predictable metastatic pattern, including potential invasion of the gastrointestinal tract. This report presents the case of a patient with ILC who developed ileocecal valve metastasis leading to cecal volvulus, a rare but critical complication. The slow dissemination of ILC delayed the diagnosis and allowed for significant tumor burden at the ileocecal junction with consequent intestinal occlusion, resulting in acute abdominal symptoms and the need for surgical intervention. Treatment strategies for ILC, which may include surgery, radiation, and systemic therapies, vary based on the tumor's stage and hormonal receptor status. In this particular case, the patient underwent an emergent right hemicolectomy with subsequent treatment with fulvestrant. This case highlights the importance of vigilant monitoring for unusual metastatic sites in ILC and underscores the need for comprehensive treatment approaches tailored to individual patient profiles.

## Introduction

Lobular breast cancer, more precisely, invasive lobular carcinoma (ILC), is the second most common form of invasive breast cancer, after invasive ductal carcinoma, accounting for 10-15% of cases [1]. It is more common in women, particularly those over 50 years of age, and has been observed to have a slightly increasing incidence over the years [1].

This type of cancer is characterized by cancerous cells originating in the lobules of the mammary glands and one of its distinctive features is the propensity for slow and insidious dissemination. ILC exhibits a less predictable pattern of distant spread. It commonly metastasizes to bone, liver, and lungs, but also can invade unusual areas such as the gastrointestinal (GI) tract (including the stomach, small intestine, and colon), as well as the ovaries and peritoneum [1].

According to the American Joint Committee on Cancer, ILC treatment may include surgery, radiation therapy, systemic therapy (hormonal therapy and/or chemotherapy), and targeted therapy, depending on the staging and hormonal status of the tumor.

## Case presentation

A 72-year-old female patient presented to the Emergency Department with nonspecific GI symptoms such as abdominal pain, distension, and alterations in bowel habits (alternating between diarrhea and constipation) in the past three months. She had a history of ILC (TNM (Tumour, Node, Metastasis): T3, N0) diagnosed and treated five years prior with total mastectomy with sentinel node biopsy, adjuvant chemotherapy, and hormonal therapy with tamoxifen, The patient was under regular follow-up with Oncology and showed no evidence of local recurrence or distant metastasis.

Due to her complaints, the oncologist requested a colonoscopy which revealed a suspicious lesion in the ileocecal valve, and a biopsy was performed. Histopathological results confirmed the presence of cancerous cells consistent with ILC (positive for CK7, GATA3 (GATA Binding Protein 3), and epithelial cadherin (E-cadherin), G2, estrogen receptor (ER) 100%, progesterone receptor (PR) 0%, human epidermal growth factor receptor 2 (HER2) negative, and Ki67 10%. She was initiated on fulvestrant promptly. (Fulvestrant is a drug used in positive metastatic breast cancer in postmenopausal women with disease progression following anti-estrogen therapy. It is an ER antagonist with no agonist effects, which works both by down-regulating and by degrading the ER.) As part of the regular follow-up, the patient underwent a whole-body CT scan that revealed no other distant metastases.

**Figure 1:**
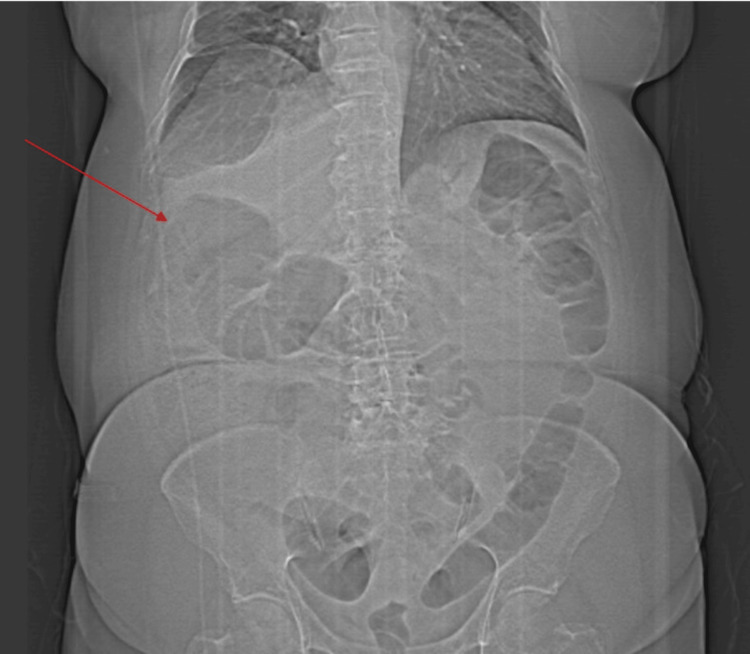
Abdominal X-ray demonstrates "coffee-bean sign"; the cecal volvulus is highlighted (red arrow).

**Figure 2:**
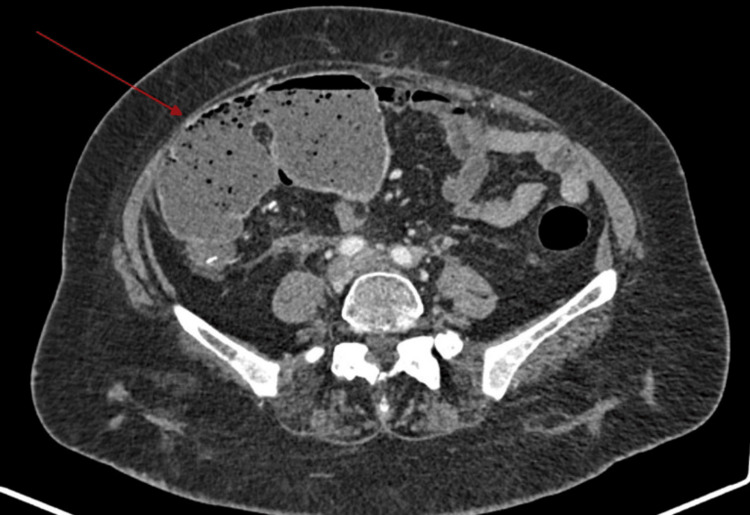
Abdomino-pelvic CT (axial view) demonstrating cecal volvulus (red arrows)

**Figure 3:**
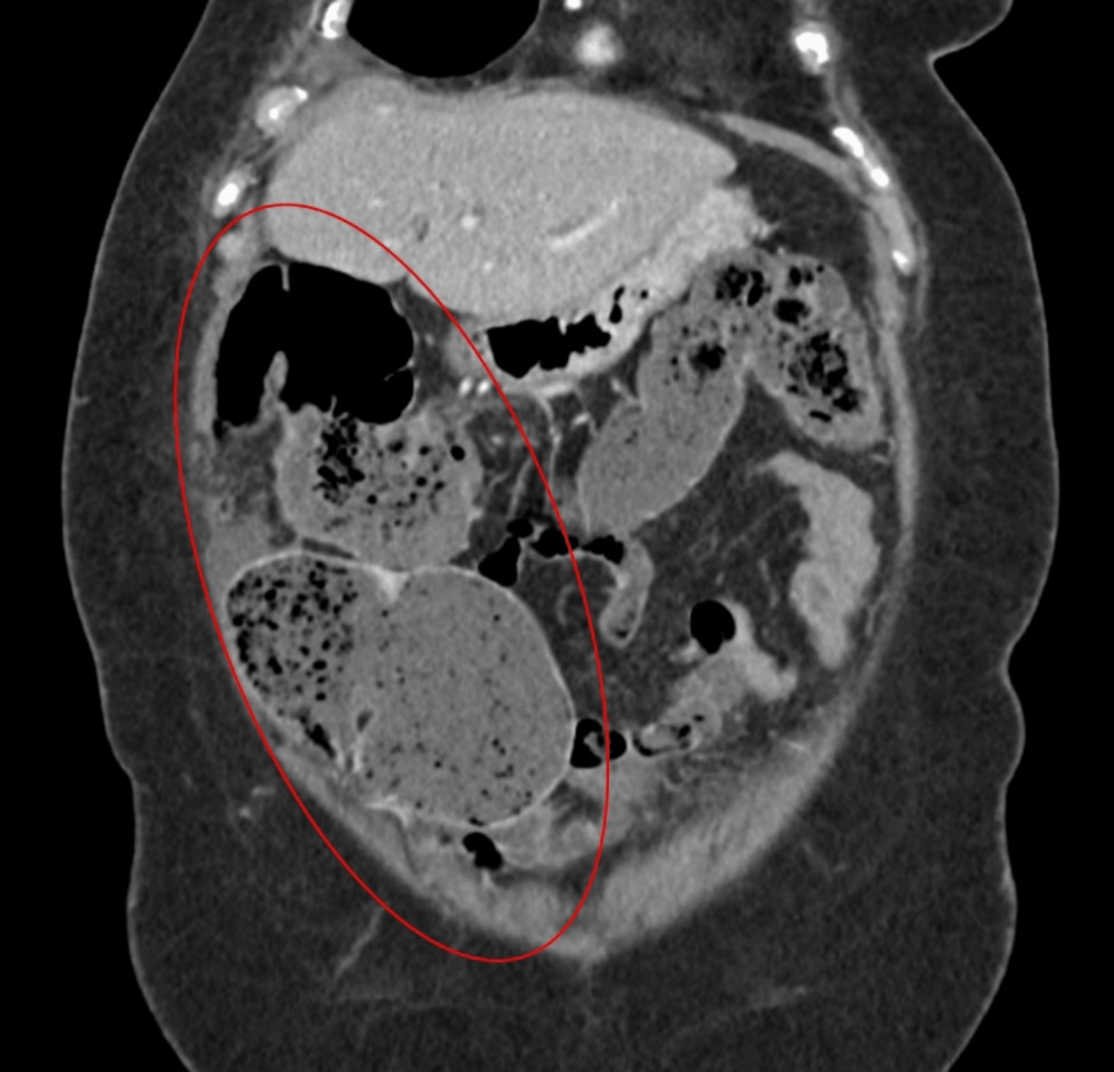
Abdomino-pelvic CT (coronal view) demonstrating cecal volvulus (red oval)

Three months after diagnosis of ileocecal valve metastasis, the patient presented to the emergency department, , with abdominal distension, constipation, and guarding. A plain abdominal X-ray was performed that revealed signs of bowel obstruction with a dilated gas-filled small bowel and a rounded focal collection of air-distended bowel with haustral creases in the upper left quadrant, increasing the suspicion for cecal volvulus (Figure 1). Later, an abdominopelvic CT confirmed the diagnosis of intestinal occlusion due to cecal volvulus (Figures 2, 3).

Emergency surgery was required and a right hemicolectomy with side-to-side ileotransverse mechanical anastomosis was performed to resect the affected intestinal segment and remove the metastatic lesion in order to relieve the symptoms. On abdominal exploration, implants of peritoneal carcinomatosis were noted on both diaphragmatic cupulas. The histopathological result was positive for metastasis from lobular carcinoma (from the primary tumor), ER was 100%, PR 0%, and HER2 negative. The patient had a successful postoperative recovery and subsequently underwent adjuvant chemotherapy to control any residual disease. She is under regular follow-up to assess treatment response and monitor for recurrence.

## Discussion

The occurrence of metastasis in the ileocecal valve from ILC is a rare event and the complication of cecal volvulus is even more uncommon [2]. This condition presents significant diagnostic and therapeutic challenges. Early diagnosis is essential for effective treatment but can be challenging due to the nonspecific presentation of gastrointestinal symptoms [3,4].

In this case, the location of the metastasis caused a cecal volvulus conditioning an intestinal occlusion, leading to a need for emergency surgery, the right hemicolectomy. Emergency surgery is often necessary to relieve intestinal obstruction and treat the acute complication. Other surgical options may include ileocecal resection or an ileocolic bypass, depending on the clinical situation. A multidisciplinary approach involving surgeons, oncologists, and gastroenterologists plays a crucial role in managing these complex cases [5].

## Conclusions

This case report illustrates the complexity of metastasis from ILC given its, sometimes, unpredictable pattern, especially when this metastasis leads to severe complications such as cecal volvulus. Early diagnosis, emergency surgery, and adjuvant treatment are key elements in the effective management of these rare cases. Furthermore, it underscores the importance of continuous surveillance in a patient with a history of lobular breast carcinoma, even after successful initial treatment, to identify and treat any recurrences or metastases early. More research is needed to better understand the underlying mechanisms of this form of metastasis and develop more effective early detection and treatment strategies as well as including possible modifications to the surveillance/monitoring protocols for ILC patients.
